# *AARS2* leukoencephalopathy: A new variant of mitochondrial encephalomyopathy

**DOI:** 10.1002/mgg3.582

**Published:** 2019-01-31

**Authors:** Yi Tang, Qi Qin, Yi Xing, Dongmei Guo, Li Di, Jianping Jia

**Affiliations:** ^1^ Innovation Center for Neurological Disorders Department of Neurology Xuan Wu Hospital Capital Medical University Beijing China; ^2^ Center of Alzheimer's Disease Beijing Institute for Brain Disorders Beijing Key Laboratory of Geriatric Cognitive Disorders Neurodegenerative Laboratory of Ministry of Education of the People's Republic of China Beijing China

**Keywords:** *AARS2* leukoencephalopathy, alanyl‐transfer (t)RNA synthetase 2, leukoencephalopathies, mitochondrial encephalomyopathy

## Abstract

**Background:**

Mutations in the mitochondrial alanyl‐transfer (t)RNA synthetase 2 *(AARS2*,OMIM:612035) have been linked to leukoencephalopathy recently. Till now, there have been 19 cases reported so far. However, the clinical and genetic characteristics of this disease are not fully understood. We reported an adult‐onset male leukoencephalopathy patient related to novel *AARS2* gene mutations and reviewed all previous cases regarding the clinical and genetic features of *AARS2* leukoencephalopathy.

**Methods:**

The spectrum of clinical symptoms and the genetic analysis of the presented patient were identified and investigated. Besides this case, we assessed previously reported cases with *AARS2* gene mutations.

**Results:**

Here, we present a 30‐year‐old man with progressive motor deficits in the right lower limb and severe cerebellar ataxia for one year. MRI revealed extensive white matter lesions in periventricular regions and along the corticospinal tract. Genetic analysis revealed two new heterogeneous missense mutations in *AARS2*: c.179C>A and c.1703_1704del. We described the ragged red fiber (RRF) for the first time, suggesting that *AARS2*‐related leukoencephalopathy be a new variant of mitochondrial encephalomyopathy. Gradual improvement in motor function was observed with intravenous coenzyme complex treatment. We also summarized our case and all previously reported cases to provide an overview of *AARS2*‐related late‐onset leukoencephalopathy. Then, we compared clinical and neuroimaging features of *AARS2*‐related leukoencephalopathy with three other frequently diagnosed types of adult‐onset leukoencephalopathy to provide insight into diagnostic strategies.

**Conclusion:**

The characteristic MRI abnormalities and clinical symptoms described here may help to distinguish *AARS2*‐related leukoencephalopathy from other adult‐onset leukoencephalopathies. The combination of encephalopathy and myopathy strongly suggest that *AARS2*‐related leukoencephalopathy is a new variant of mitochondrial encephalomyopathy. The response to coenzyme complex will shed light on future therapy investigation.

## 1 INTRODUCTION

Adult‐onset leukoencephalopathies are a rare group of neurological diseases disorders characterized by progressive degeneration of cerebral white matter (Euro et al., 2015). They are associated with a variety of clinical phenotypes dominated by dementia, psychiatric changes, movement disorders, and upper motor neuron signs (Lakshmanan et al., 2017). The differential diagnosis of adult‐onset leukoencephalopathies is often challenging due to the high heterogeneity of etiologies (Lakshmanan et al., 2017). With advances in the genetic testing, several causative gene mutations have been shown to be responsible for the etiology of adult‐onset leukoencephalopathies (Lynch et al., 2017; Renaud, 2012). Of these gene mutations, mitochondrial alanyl‐tRNA synthetase 2 gene (*AARS2,* OMIM:612035) is a nuclear gene, accounting for AARS2 loading of alanine onto tRNA during mitochondrial translation. *AARS2* mutations can result in two very different phenotypes with different tissue involvement: some mutations particularly affect the heart (Calvo et al., 2012; Gotz et al., 2011), thus producing a fatal, early‐onset cardiomyopathy, whereas others are associated with late‐onset leukoencephalopathy without cardiac involvement, and ovarian failure also been described in female.

Leukoencephalopathy due to *AARS2* mutations is a syndrome with progressive cerebral white matter degeneration (Kaye & Moser, 2004). Since first identified in 2014, only 19 *AARS2*‐related leukoencephalopathy cases have been reported in English literature until now (Dallabona et al., 2014; Dong et al., 2018; Hamatani et al., 2016; Lee et al., 2017; Peragallo, Keller, van der Knaap, Soares, & Shankar, 2018; Szpisjak et al., 2017; Taglia et al., 2018). Owing to the low number of known cases, most clinicians have limited knowledge about the clinical presentation and neuroimaging features of *AARS2*‐related leukoencephalopathy. In addition, there are many different kinds of leukoencephalopathies, which makes a definitive diagnosis difficult.

Here, we report the first description of typical ragged red fiber (RRF) identified by muscle biopsy in a male patient with *AARS2*‐related leukoencephalopathy, suggesting that *AARS2*‐related leukoencephalopathy be a new variant of mitochondrial encephalomyopathy. We summarized the main findings of our case and all previously reported 19 cases to provide an overview of the genetic, clinical, and neuroimaging characteristics of *AARS2*‐related late‐onset leukoencephalopathy. Then, we compared clinical and neuroimaging features of *AARS2*‐related leukoencephalopathy with three other frequently diagnosed types of adult‐onset leukoencephalopathy to provide insight into diagnostic strategies.

## 2 CASE REPORT

A 30‐year‐old Chinese man was admitted with complaints of progressive motor deficits in the right lower limb for 1 year and dysarthria for 2 months. The patient's motor symptoms had developed 1 year earlier, along with an unsteady gait. Subsequently, he had gradually developed weakness and numbness of the right limbs, rigidity and aphasia, with occasional dysphagia and dysarthria. The patient had a 6‐year history of drug abuse and had taken methamphetamine on ten occasions in the previous 6 months. His symptoms were considered to be encephalopathia toxica in a local hospital and were treated with 500 mg of methylprednisolone per day followed by 30 mg prednisone per day. No improvement was observed. There was no family history of cerebellar symptoms.

Neurological examination revealed normal mental status and normal cranial nerve functions. The strength of the right lower limb was 4/5 with brisk tendon reflexes, bilateral ankle clonus, and bilateral Rossolimo and Chaddock signs. The patient also showed spastic gait and positive Romberg's sign, with slight decrease in pinprick sensation in the lower extremities. The patient was unable to perform the finger‐nose tests and rapid alternating movements.

**Figure 1:**
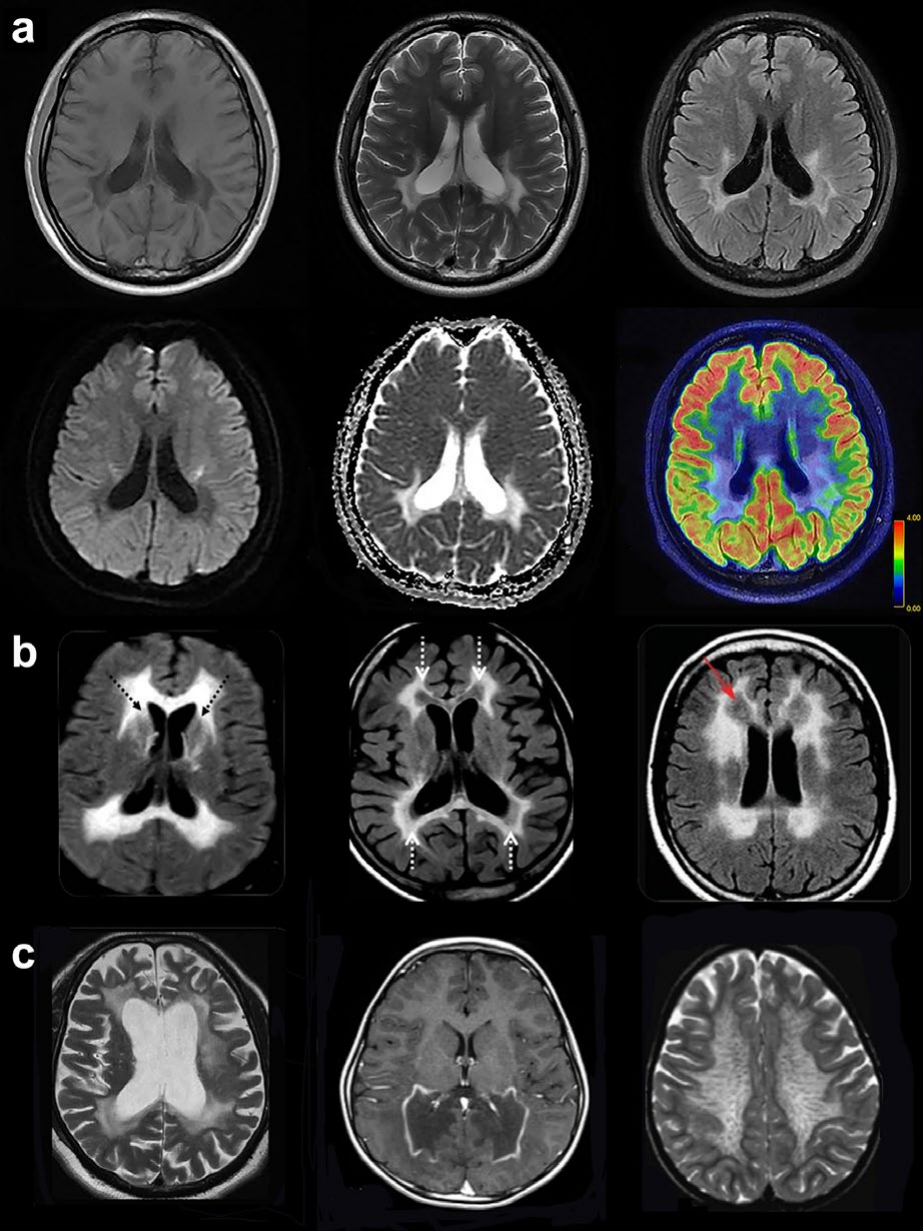
Brain MRI scans of patients with various types of leukoencephalopathy. (a) Brain MRI of P20 (the presented case) with AARS2‐related leukoencephalopathy. T1‐weighted, T2‐weighted, axial scans show symmetrical and extended white matter abnormalities at bilateral periventricular areas and corpus callosum involvement mainly in the posterior part. Fluid‐attenuated inversion recovery (FLAIR)‐weighted, axial scans present symmetrical, longitudinal, striped, and intense signals present on both sides of the corticothalamic level. The diffusion‐weighted image (DWI) and apparent diffusion coefficient (ADC) image show patchy areas of restricted diffusion in the abnormal white matter. PET MRI of the patient shows that decreased metabolism is pronounced in the white matter lesions. (b) T2‐weighted, axial scans of Patient 3 at age 33 years (left) demonstrates pronounced leukoencephalopathy at bilateral periventricular areas. Axial T2‐weighted MRI of Patient 4 at age 24 years (middle) shows that abnormal signals are found in the frontal and parietal lobe white matter and the pyramidal tract at the internal capsule. FLAIR MRI image of Patient 7 at age 30 (right) shows that the affected white matter is rarefied. (c) In a patient with ALSP (left), axial T2‐weighted MRI shows confluent slightly asymmetric frontoparietal‐predominant white matter hyperintensity, with more atrophy compared with AARS2 leukoencephalopathy. In a patient with X‐ALD (middle), axial postgadolinium contrast sequences show diffuse white matter signal T2‐weighted hyperintensity and volume loss of the posterior frontal, posterior callosal and parieto‐occipital regions. In a patient with MLD (right), an axial T2‐weighted sequence demonstrates widespread symmetrical white matter signal hyperintensity sparing the subcortical U fibers and radiating transmedullary bands

**Figure 2:**
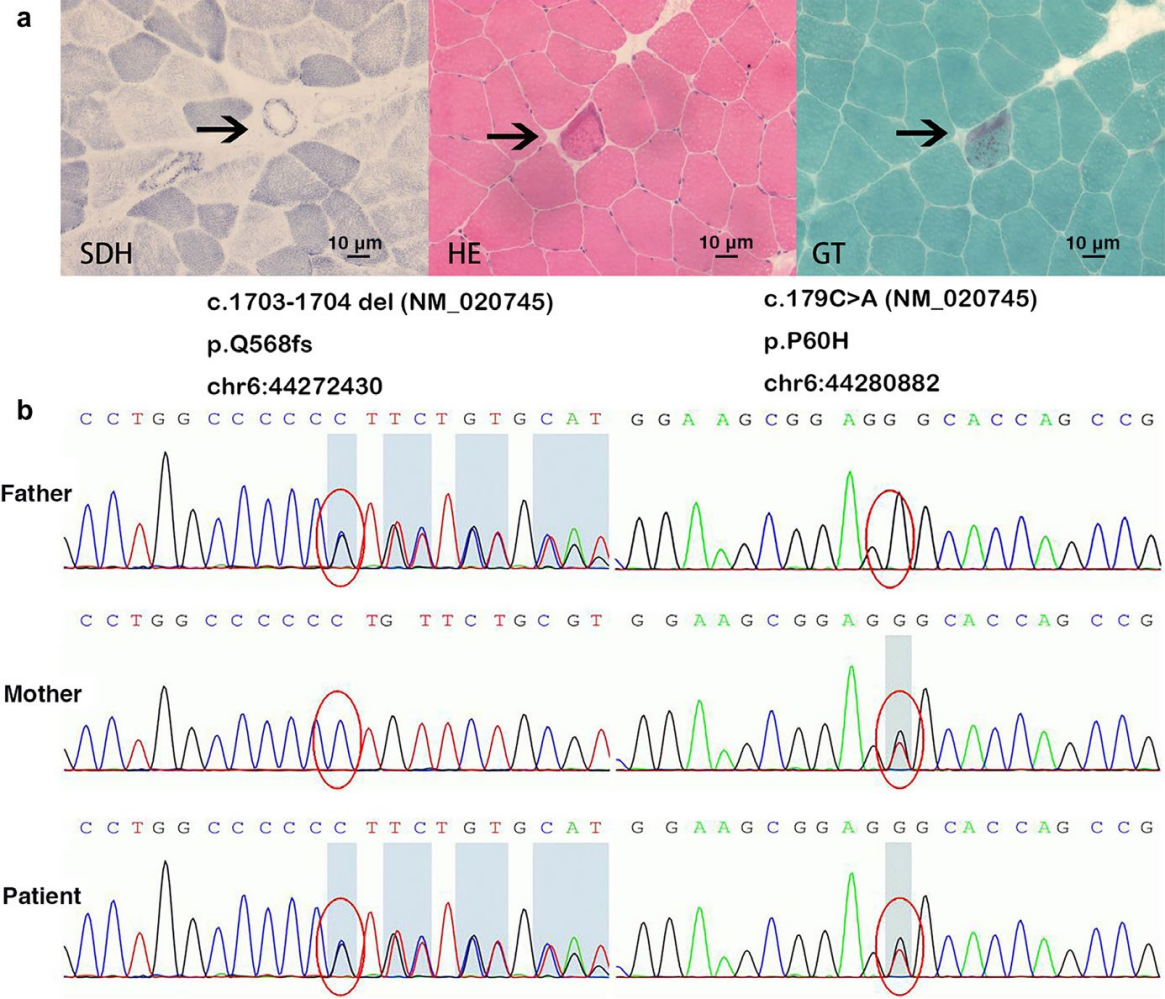
Muscle biopsy and genetic analysis of our patient. (a) Morphologic analysis of muscle biopsy, with succinate dehydrogenase (SDH) staining, hematoxylin‐eosin staining (HE) and Gomori trichrome (GT) staining. A typical RRF was indicated by arrow. (b) Sequencing of AARS2 in the presented case shows both mutations c.179C>A and c.1703_1704del

Laboratory evaluation showed normal routine studies. Examination of the cerebrospinal fluid revealed 507.7 mg/dl protein (normal range 15–45 mg/dl) and normal IgG index. Autoimmune, infectious, endocrinologic, neoplastic, and paraneoplastic screenings were unremarkable. However, serum levels of alanine aminotransferase and lactic acid (instant state, resting state, 1 min, and 10 min) were all increased. Brain magnetic resonance imaging (MRI) showed abnormal signals in the bilateral periventricular white matter, the posterior part of the corpus callosum and symmetrically along the corticospinal tract without gadolinium enhancement. In addition, a thin posterior corpus callosum, enlarged lateral ventricle, and widened bilateral parietal sulcus were demonstrated. The diffusion‐weighted image (DWI) shows patchy areas of restricted diffusion in the abnormal white matter, confirmed by low signal of the corresponding areas on the apparent diffusion coefficient (ADC) map. The restricted diffusion areas exist for more than 1 year. PET‐MRI showed hypometabolism in the posterior part of the cerebral white matter (Figure 1a). A typical RRF was identified in muscle biopsy for the first time (Figure 2a).

Targeted gene sequencing of *AARS2* identified two variations: c.179C>A and c.1703_1704del (Figure 2b). These two mutations have not been previously described, and they were not found in the ExAC database (http://exac.broadinstitute.org/gene). The patient was compound heterozygote for these pathogenic mutations, which were transmitted maternally and paternally, respectively (Figure 2b).

The treatment was generally supportive. After the application of intravenous coenzyme complex 200 U one time per day for 10 days, the patient showed gradual improvement in motor function. He could walk 1,000 m alone and remained stable on six‐month follow‐up. However, the patient showed rapidly deterioration of cerebellar ataxia and motor deficits in 1 year.

## 3 LITERATURE REVIEW

To date, *AARS2* mutations have been reported in 19 patients with late‐onset leukoencephalopathy. To provide an overview of the clinical characteristics of *AARS2*‐related late‐onset leukoencephalopathy, we searched the MEDLINE database to October 2018 using the terms “*AARS2*” or “alanyl‐tRNA synthetase 2 gene” and reviewed all identified articles and articles referenced therein, including non‐English articles (Dallabona et al., 2014; Dong et al., 2018; Hamatani et al., 2016; Lee et al., 2017; Peragallo et al., 2018; Szpisjak et al., 2017; Taglia et al., 2018). We identified 19 previous cases and combined them with the current case to provide a summary of the genetic, clinical, and neuroimaging features of *AARS2*‐related leukoencephalopathy.

### 3.1 Clinical features

The detailed clinical features of the case presented here and the previously reported patients (9 males and 11 females) are summarized in Table 1. The patients had adult‐onset signs of neurologic deterioration with an average age of onset of 27.3 years. All 20 patients exhibited rapid deterioration after onset. Psychiatric symptoms, such as changes in personality, delusions, anxiety and depression, usually preceded neurological symptoms. The neurological dysfunctions are composed of motor deterioration, including cerebellar ataxia and pyramidal signs, and progressive cognitive decline with features of frontal lobe dysfunction, such as poor attention, inactivity, and other neurological changes. Among these symptoms, cognitive decline was the most frequent (18 patients [90%]). Pyramidal signs (15 patients [75%]) and cerebellar ataxia (13 patients [65%]) were also prominent clinical features. Psychiatric dysfunction, such as anxiety and depression (11 patients [55%]), was often observed. All women with the condition had primary or secondary amenorrhea related to ovarian failure. General physical examination usually revealed no abnormalities and no evidence of cardiac dysfunction.

**Table 1:** Clinical and genetic characteristics of patients with *AARS2‐*mutations reported in literature

Case number	Sex	Age at onset (years)	First neurological symptoms	Cerebellar symptoms	Cognitive impairment	Psychiatric symptoms	Pyramidal signs	Ovarian failure	AARS2 mutation	Amino acid change	Reference
P1	Female	15	Gait ataxia, cognitive decline, tremor	+	+	+	−	+	c.149T>G; c.1561C>T	p.Phe50Cys; p.Arg521a	8
P2	Male	7	Ataxia	+	+	−	+	Male	c.2893G>A; c.1213G>A	p.Gly965Arg; p.Glu405Lys	8
P3	Female	33	Cognitive decline and depression	+	+	+	+	+	c.1609C>T & c.2350del; c.595C>T	p.Gln537 & p.Glu784Serfs*9; p.Arg199Cys	8
P4	Female	15	Tremor	+	+	+		+	c.230C>T; c.595C>T	p.Ala77Val; p.Arg199Cys	8
P5	Female	40	Cognitive decline and depression	−	+	+	−	+	c.595C>T; c.390_392del	p.Arg199Cys; p.Phe131del	8
P6	Female	22	Spastic paraparesis and depression	+	−	+	+	+	c.595C>T; c.2611dup	p.Arg199Cys; p.Thr871Asnfs*21	8
P7	Female	30	Cognitive decline and psychosis	−	+	+	+	+	c.1145C>A; c.2255 + 1G> A*	p.Thr382Lys	9
P8	Male	27	Psychosis	+	+	+	+	Male	c.578T>G; c.595C>T	p.Leu193a; p.Arg199Cys	10
P9	Female	40	Anxiety and cognitive decline	−	+	−	+	+	c.1041‐1G>A*; c.595C>T	p. Arg199Cys	11
P10	Male	Late 30s	Cognitive decline	−	+	−	−	Male	c.1188G>A*; c.1709delG	p. Gly570Afs*21	11
P11	Male	Mid 20s	Rapid motor decline	+	+	+	+	Male	c.1188G>A*; c.1709delG	p. Gly570Afs*21	11
P12	Male	15	Cognitive decline and psychosis	+	+	+	+	Male	c.892_894del; c.2234_2235 del	p.298_298delGln; p.Ser745Cysfs*60	11
P13	Male	Mid 40s	Right upper limb dystonia	−	+	+	+	Male	c.595C>T (homozygous)	p. Arg199Cys	11
P14	Male	17	Tremor	+	+	+	+	+	c.2265dupA; c.650C>T	p.Arg756 fs; p.Pro217Leu	13
P15	Female	44	Gait ataxia and cognitive decline	+	+	−	+	+	c.595C>T; c.390_392del	p.Arg199Cys; p.Phe131del	14
P16	Female	32	Cognitive decline	−	+	−	−	+	c.595C>T; c.236T>A	p.Arg199Cys; p.Met79Lys	14
P17	Female	23	Ataxia and spastic gait	+	−	−	+	+	c.595C>T; c.2611_2612 insA	p.Arg199Cys; p.Thr871Asnfs	14
P18	Female	33	Ovarian failure and cognitive decline	−	+	−	+	+	c.963C>A; c.452T>C	Tyr321*; p.Met151Thr	12
P19	Male	35	Dystonia	+	+	−	+	Male	c.963C>A; c.452T>C	Tyr321*; p.Met151Thr	12
P20	Male	29	Axatia	+	+	−	+	Male	c.179C>A; c.1703_1704del	p.Arg199Cys; p.Val730Met	Presented case

### 3.2 Genetic analysis

Genetic analysis revealed that in all 20 cases the mutations followed autosomal recessive inheritance. Nineteen patients were compound heterozygotes for *AARS2* mutations and only one male patient was homozygous. The pathogenic gene mutations were derived from the paternal and maternal side. All nucleotide and amino acid changes are summarized in Table 1. Among these nucleotide changes, c.595C>T was the most common (nine patients). The most prominent amino acid changes were p.Arg199Cys. P20 (the present case) carried two new heterogeneous missense mutations, c.179C>A and c.1703_1704del.

### 3.3 Neuroimaging features

The MRI abnormalities of the 20 patients are summarized in Table 2. All patients exhibited T2‐W hyperintense, T1‐W hypointense white matter signal abnormalities. Typically, the abnormalities were predominantly present in the frontal and parietal periventricular and deep white matter, sparing a segment of white matter in between. White matter structures were affected in a tract‐like manner, with involvement of interhemispheric connections through the corpus callosum and descending pathways. The cases with involvement of the corpus callosum may show either a single lesion in the splenium or the entire corpus callosum in an inhomogeneous manner. Among the other patients, the lesion involved the entire corpus callosum in an inhomogeneous manner. The abnormalities were confined to white matter structures and were progressive over time. No gadolinium enhancement was observed. Cerebellar atrophy was variable, and cerebral atrophy, if present, was mild.

**Table 2:** Clinical examinations of patients with *AARS2*‐mutations

Case number	MRI abnormalities					Cardiac ultrasound	ECG	Muscle biopsy	Plasma lactate level	Sex hormone levels
	White Matter Rarefaction	Corticospinal Tracts	Corpus Callosum	Atrophy	U‐fibers					
P1	+	+	+	+	−	Normal	Normal	Isolated COX deficiency	Normal	Low estradiol, FSH & LH↑
P2	+	+	+	−	−	n.i.	Normal	n.i.	Normal	n.i.
P3	+	+	+	−	+	n.i.	Normal	n.i.	Normal	Low estradiol, FSH & LH↑
P4	+	+	−	−	−	n.i.	n.i.	n.i.	Normal	FSH & LH↑
P5	+	+	−	−	−	n.i.	n.i.	n.i.	n.i.	FSH, LH and prolactin↑
P6	+	+	−	−	−	n.i.	Normal	Isolated COX deficiency	n.i.	low estradiol, normal FSH &LH
P7	+	+	+	+	+	Normal	Normal	Normal	n.i.	Normal
P8	−	+	−	−	−	Normal	Normal	n.i.	n.i.	Normal
P9	−	+	+	+	−	n.i.	n.i.	n.i.	Increased	Low estradiol, FSH & LH↑
P10	+	+	+	−	−	Normal	Normal	n.i.	Normal	Normal
P11	+	+	+	−	−	Normal	Normal	n.i.	Normal	Normal
P12	−	+	+	+	−	Normal	Normal	n.i.	Normal	Normal
P13	+	+	+	−	−	Normal	Normal	n.i.	Normal	Normal
P14	+	+	+	+	−	Normal	Normal	n.i.	Normal	Normal
P15	+	+	+	−	+	n.i.	Normal	n.i.	n.i.	n.i.
P16	+	+	−	+	−	n.i.	n.i.	n.i.	n.i.	n.i.
P17	+	+	−	−	−	n.i.	n.i.	n.i.	n.i.	n.i.
P18	+	+	+	−	−	Normal	n.i.	n.i.	n.i.	n.i.
P19	+	+	+	−	−	Normal	n.i.	n.i.	n.i.	n.i.
P20	+	+	+	+	+	Normal	Normal	Typical RRF	Increased	Normal

### 3.4 Muscle biopsy and cardiac function analysis

Morphologic analysis of muscle biopsy and cardiac function analysis of the patients with *AARS2* mutations are summarized in Table 2. Cardiac ultrasonography and electrocardiogram revealed no evidence of cardiac dysfunction. Patients P1, P6, P7, and P20 (the presented case) underwent skeletal muscle biopsy to further investigate possible mitochondrial defects. Patients P1 and P6 showed isolated cytochrome c oxidase deficiency. Our group identified a typical RRF in the patient with *AARS2*‐related leukoencephalopathy for the first time.

### 3.5 Laboratory findings

The laboratory findings, including the results of routine hematology and biochemistry tests, were normal among all 20 patients (Table 2). None of the patients had elevated CSF lactate levels; however, patients P9 showed increased plasma levels of lactate. Most women with ovarian failure showed low estradiol levels and high levels of follicle‐stimulating hormone (FSH) and luteotropic hormone (LH).

## 4 DISCUSSION

Herein, we present the case of a 30‐year‐old man with progressive motor deficits in the right lower limb and severe cerebellar ataxia for 1 year. MRI revealed extensive white matter lesions in bilateral periventricular regions and the posterior part of corpus callosum and along the corticospinal tract. Genetic analysis revealed two new heterogeneous missense mutations in *AARS2*: c.179C>A and c.1703_1704del. The serum lactic acid level was elevated. It is noteworthy that this is the first reported case of *AARS2*‐related leukoencephalopathy with a typical RRF identified by muscle biopsy.

As *AARS2*‐related encephalopathy is a newly reported encephalopathy, clinicians have scant knowledge about the clinical presentation and neuroimaging features. By reviewing all published cases, the following findings are strongly indicative of *AARS2*‐related leukoencephalopathy: late‐onset and rapidly progressing encephalopathy; cerebellar ataxia and cognitive decline; white matter signal abnormalities in periventricular regions, posterior part of the corpus callosum and symmetrically along the corticospinal tract. In the presence of these symptoms, the diagnosis should be confirmed by genetic analysis.

Adult onset leukoencephalopathy is a category of neurological disorders with high heterogeneity. It is always difficult to make definite diagnosis, even for experienced neurologists. Therefore, we compared *AARS2*‐related leukoencephalopathy with three other types of common adult‐onset leukoencephalopathies to provide insight into diagnostic strategies, including leukoencephalopathy with axonal spheroids and pigmented glia (ALSP) (Lynch et al., 2016), X‐linked adrenoleukodystrophy (X‐ALD) (Mirabella, Di Giovanni, Silvestri, Tonali, & Servidei, 2000), and metachromatic leukodystrophy (MLD) (Taylor et al., 2014) (Table 3). ALSP is the most common adult‐onset leukoencephalopathy caused by autosomal dominant mutations in the colony‐stimulating factor receptor 1 (*CSF1R*) gene. Mutations in the *CSF1R* gene occur in the tyrosine kinase domain of the CSF1R, which is primarily expressed in the microglia in the CNS, and result in microglial dysfunction in ALSP. X‐ALD is caused by mutations in the ABCD1 gene encoding the adrenoleukodystrophy protein (ALDP). X‐ALD is an X‐linked disorder that results from a deficient very‐long‐chain fatty acid transport protein on the surface of the peroxisome. The adult‐onset form accounts for 5% of all X‐ALD patients. Metachromatic leukodystrophy (MLD) is an autosomal recessive lysosomal disorder owing to *ARSA* gene mutations resulting in deficiency of the enzyme arylsulfatase A (ASA). The adult form accounts for approximately 20% of all MLD cases. ALSP has a relatively late onset, typically occurring in the fifth decade, as opposed to *AARS2*, for which the disease onset tends to occur in the third decade of life. Men and women with adult‐onset X‐ALD may remain asymptomatic into the fourth and sixth decade of life, respectively. The age of MLD's onset can be up to 70 years. The course after deterioration onset of ALSP and AARS2 is rapid; however, disease progression of X‐ALD and MLD is generally slower, with death occurring after decades. Cognitive and motor symptoms are the most common symptoms overall in all four leukoencephalopathies. Patients with ALSP and AARS2 typically present with cognitive impairment and gait problems first, then develop spasticity and ataxia. Adult‐onset cerebral ALD usually presents with psychiatric features followed by cognitive impairment, ataxia, movement disorders, and peripheral neuropathy. Adrenal insufficiency is also frequently associated with the condition. The initial symptoms of MLD are often psychiatric and are followed by motor symptoms, including spastic paraparesis and cerebellar ataxia, then intellectual and cognitive decline. MLD patients often experience other symptoms late in life, including optic nerve atrophy and peripheral neuropathy. All female patients with *AARS2* mutation presented with ovarioleukodystrophy and ovarian failure, a feature not seen in other leukoencephalopathies.

**Table 3:** Clinical features of patients with *AARS2*‐mutations and other common types adult‐onset leukoencephalopathies

Disorder	Age of onset	Prevalence	Inheritance	Affected gene(s)	Cognitive involvement	Movement disorder	Pyramidal weakness/spasticity	Cerebellar ataxia	Peripheral neuropathy	Psychiatric symptoms	Visual involvement	Primary/secondary amenorrhea	Adrenal insufficiency	Hyper‐pigmentation
AARS2‐L	Mean 20 years	20 reported cases	AR	AARS2	+	+	+	+	−	+	−	+	−	−
ALSP	Mean 40 years	52 reported cases	AD	CSFR1	+	+	+	+	−	−	−	−	−	−
X‐ALD	Childhood to adulthood	Up to 40/million, 5% adult onset	X linked	ABCD1	−	+	+	−	−	+	−	−	+	+
MLD	Up to 70 years	2/million, 20% adult onset	AR	ARSA	+	+	+	+	+	+	+	−	−	−

Brain MRI of all these leukoencephalopathies show confluent patterns of white matter lesions (van der Knaap, Breiter, Naidu, Hart, & Valk, 1999; Schiffmann & van der Knaap, 2009) (Table 4). However, the predominant pattern is different. In *AARS2*‐related leukoencephalopathy and ALSP, white matter changes are typically seen in the frontal and parietal periventricular region, whereas white matter abnormalities are usually seen in occipital regions in X‐ALD and frontal regions in MLD. Corpus callosum involvement is present in all diseases. Cerebral and cerebellar atrophy are prominent in ALSP and MLD and are disproportionately mild in *AARS2*. Spinal cord atrophy may develop in X‐ALD. The distribution of white matter lesions is symmetric in AARS2 and MLD and asymmetric in ALD and ALSP. Each leukoencephalopathy has a unique pattern of MRI‐abnormalities. ALSP occasionally shows calcifications in the frontal periventricular white matter. In cerebral ALD, contrast enhancement at the periphery of the white matter lesions is characteristic. The subcortical U‐fibers are typically spared in MLD. *AARS2*‐related leukoencephalopathy is associated with white matter rarefaction, which suppresses on a FLAIR image, a feature not seen in other leukoencephalopathies.

**Table 4:** Neuroimaging features of AARS2‐related leukoencephalopathy and three other common adult‐onset leukodystrophies

Disorder	Pattern of white matter involvement	Findings in the corpus callosum	Atrophy	Symmetry	Unique MRI features
AARS2‐L	Predominantly frontoparietal periventricular	Atrophy in the posterior part	Mild cerebral and cerebellar atrophy	Symmetric	White matter rarefaction
ALSP	Predominantly frontoparietal periventricular	Thinning	Prominent cerebral atrophy	Asymmetric	Calcifications
X‐ALD	Predominantly parieto‐occipital	Atrophy in the splenium	Spinal cord atrophy	Asymmetric	Contrast enhancement
MLD	Predominantly frontal periventricular	Thinning	Cerebral atrophy	Symmetric	Spared U fibers

The pathogenesis of *AARS2*‐related encephalopathy is not clear yet. Here, for the first time we reported a typical RRF identified by muscle biopsy in a patient with *AARS2*‐related leukoencephalopathy, which suggests that *AARS2*‐related leukoencephalopathy might be a new variant of mitochondrial encephalomyopathy. Mitochondrial encephalomyopathy is a heterogeneous group of disorders with various biochemical defects in the respiratory chain (McFarland, Taylor, & Turnbull, 2010; Shoubridge, 1998). Mutation of *AARS2*, which is also a kind of mtARS mutation (Diodato, Ghezzi, & Tiranti, 2014), may affect mitochondrial protein synthesis and functioning of the respiratory chain, and thus might cause a disorder of energy metabolism. Mitochondrial encephalomyopathy can lead to a wide range of nervous system and muscle disorders (Moulinier, Ripp, Castillo, Poch, & Sissler, 2017). The diagnostic workup routinely includes skeletal muscle biopsies and genetic studies (Bernier et al., 2002; Bourgeois & Tarnopolsky, 2004). Isolated cytochrome c oxidase (COX) deficiency and typical RRF could be shown in the skeletal muscle biopsy of mitochondrial encephalomyopathy (Holt, Harding, & Morgan‐Hughes, 1988; Shy, 1964). COX is a multimeric inner mitochondrial membrane enzyme formed by subunits encoded by nuclear or mitochondrial genomes. The RRF is the histological hallmark of abnormal mitochondria proliferation in muscle fibers. Identification of any RRF in an individual aged <30 years is indicative of mitochondrial disease and can be regarded as a diagnostic indicator of myopathy. All of these findings strongly suggest that *AARS2*‐related leukoencephalopathy is a new variant of mitochondrial encephalomyopathy.

Genetic analysis demonstrated that the mutations in all 20 cases followed autosomal recessive inheritance, and 19 patients were compound heterozygotes for *AARS2* mutations. To date, *AARS2* mutations have been reported among patients with infantile mitochondrial hypertrophic cardiomyopathy with early fatal outcomes, and patients with late‐onset leukoencephalopathy. Therefore, the question arises as to why *AARS2* mutations cause two very different subtypes with distinct systemic involvement. Euro et al. (Euro et al., 2015) have provided an elegant explanation for this phenomenon. In addition to the aminoacylation domain and the C‐terminal domain (Guo et al., 2009), AARS2 also has an editing domain that can deacylate mischarged amino acids. None of the missense mutations identified in patients with adult‐onset leukoencephalopathy were in the editing domain, whereas at least one c.1774C>T mutation in the editing domain was present in patients with infantile‐onset cardiomyopathy (Scheper et al., 2007). The difference in the location of *AARS2* missense mutations leads to different aminoacylation activity, such that cardiomyopathy results from a severe decrease in aminoacylation activity, and leukoencephalopathy results from only a moderate decrease. The location of the mutations in P20 (the present case) is consistent with this hypothesis.

## 5 CONCLUSIONS

In conclusion, we report an *AARS2*‐related leukoencephalopathy case with typical RRF for the first time. On the basis of reviewing all known cases, the following findings are strongly indicative of *AARS2*‐related leukoencephalopathy: late‐onset and rapidly progressing encephalopathy; progressive cognitive decline; cerebellar ataxia; white matter signal abnormalities in the frontal and parietal periventricular regions, posterior part of the corpus callosum and symmetrically along the corticospinal tract; and amenorrhea secondary to ovarian failure among affected women. In the presence of these symptoms, the diagnosis should be confirmed by genetic analysis. Finally, on the basis of the combination of encephalopathy, increased plasma lactate level, isolated cytochrome c oxidase deficiency and typical RRF in muscle biopsy, we propose that *AARS2*‐related leukoencephalopathy is a new variant of mitochondrial encephalomyopathy. Currently, there are no treatment options for *AARS2* leukoencephalopathy. The good response to intravenous coenzyme complex in our patient will shed light on future treatment development.

## CONFLICT OF INTEREST

None declared.
